# Untargeted Metabolome Analysis of Alcohol-Exposed Pregnancies Reveals Metabolite Differences That Are Associated with Infant Birth Outcomes

**DOI:** 10.3390/nu14245367

**Published:** 2022-12-17

**Authors:** Julie M. Hasken, Marlene M. de Vries, Anna-Susan Marais, Philip A. May, Charles D. H. Parry, Soraya Seedat, Sandra M. Mooney, Susan M. Smith

**Affiliations:** 1 Nutrition Research Institute, University of North Carolina at Chapel Hill, Kannapolis, NC 28081, USA; 2 Department of Psychiatry, Faculty of Medicine and Health Sciences, Stellenbosch University, Tygerberg 7602, South Africa; 3 Department of Nutrition, University of North Carolina at Chapel Hill, Kannapolis, NC 28081, USA; 4 Center on Alcohol, Substance Abuse, and Addictions, University of New Mexico, Albuquerque, NM 87131, USA; 5 Alcohol, Tobacco, and Other Drug Research Unit, South African Medical Research Council, Cape Town 7760, South Africa

**Keywords:** metabolomics, FASD, prenatal alcohol exposure, infant birth measures

## Abstract

Prenatal alcohol exposure can produce offspring growth deficits and is a leading cause of neurodevelopmental disability. We used untargeted metabolomics to generate mechanistic insight into how alcohol impairs fetal development. In the Western Cape Province of South Africa, 52 women between gestational weeks 5–36 (mean 18.5 ± 6.5) were recruited, and they provided a finger-prick fasting bloodspot that underwent mass spectrometry. Metabolomic data were analyzed using partial least squares-discriminant analyses (PLS-DA) to identify metabolites that correlated with alcohol exposure and infant birth outcomes. Women who consumed alcohol in the past seven days were distinguished by a metabolite profile that included reduced sphingomyelins, cholesterol, and pregnenolones, and elevated fatty acids, acyl and amino acyl carnitines, and androsterones. Using PLS-DA, 25 of the top 30 metabolites differentiating maternal groups were reduced by alcohol with medium-chain free fatty acids and oxidized sugar derivatives having the greatest influence. A separate *ortho*-PLS-DA analysis identified a common set of 13 metabolites that were associated with infant length, weight, and head circumference. These included monoacylglycerols, glycerol-3-phosphate, and unidentified metabolites, and most of their associations were negative, implying they represent processes having adverse consequences for fetal development.

## 1. Introduction

Pregnancy is a period of dynamic physiological changes for the expectant mother as she adapts to support fetal growth and her own nutrition and metabolic needs. Maternal adaptations are reflected, in part, in a blood metabolite profile that includes changes in certain amino acid derivatives, acyl-carnitine, fatty acids, phospholipids, and sphingomyelins as the pregnancy progresses [1,2]. Failure to implement these adaptive changes can adversely affect fetal outcome. This may include impairments in maternal metabolism that limit nutrient availability for the growing fetus. The fetus adapts to maternal malnutrition by redistributing blood flow, increasing catabolic hormone production (e.g., cortisol, catecholamines, and glucocorticoids), and decreasing anabolic hormone production (e.g., insulin, insulin-like growth factor-1, and growth hormone) [3,4]. This can result in fetal growth suppression, dysregulated utilization of glucose, and persistent alterations in fetal metabolic function and tissue morphology, even if adequate nutrition is available postpartum [3,4]. Maladaptive maternal and fetal metabolic changes have been reported in complicated pregnancies [5], preeclampsia [6,7], preterm births [8,9,10], and in preclinical models of prenatal alcohol exposure (PAE) [11,12].

The functional consequence of PAE is fetal alcohol spectrum disorders (FASD), which have been found to affect 1 to 9% in regional samples and are conservatively estimated to affect 6.5% of children in the United States [13]. Worldwide, the FASD prevalence estimate is 7.7 per 1000 [14], with the highest documented prevalence reported in the Western Cape Province of South Africa where 18–30% of children fall within the FASD continuum [15,16,17,18]. Hallmark characteristics of FASD include somatic growth deficits, behavioral and cognitive deficits that affect domains of learning, memory, and attention, and a unique craniofacial appearance [19,20,21]. The severity of these growth and neurodevelopmental impairments have been partially linked to the quantity, frequency, and timing of gestational alcohol exposure [22] as well as the pregnant woman’s ability to metabolize alcohol [23]. An additional modifier of the severity of PAE on the fetus is maternal nutrition. The mixed-race ancestry (“Coloured”) population in the Western Cape Province has both a high FASD prevalence and also experiences a broad range of nutrient insufficiencies [24,25]. Alcohol disrupts the metabolism of multiple nutrients including choline and other methyl donors [26], iron [27,28,29], zinc [30], and docosahexaenoic acid (DHA) [31]. Such limitations in nutrient intake and utilization could have profoundly negative consequences for perinatal growth and development.

Untargeted analysis of metabolite production and/or utilization can provide insights into underlying mechanisms for alcohol’s teratogenicity, and could also inform the development of biomarkers to identify the severity of risk for individual pregnancies [32]. Previously in a targeted metabolomic analysis of non-pregnant adults, alcohol consumption affected sphingolipid and glycerophospholid metabolism through increased acid sphingomyelinase activity resulting in ceramide accumulation and a reduction in sphingomyelins [33]. Our prior untargeted metabolomic analyses in a preclinical model of FASD identified enrichments in gut microbiota-dependent metabolites, reduced maternal hepatic glucose content, and enriched essential amino acid catabolites, urea content, and free fatty acids [11,12]. To our knowledge, this approach has yet to be applied in a clinical context.

The purpose of this study was to use an untargeted analytical approach to identify metabolic differences between pregnant women who consumed alcohol in the previous 7 days relative to pregnant women who abstained in the previous 7 days and determine which maternal metabolites were associated with infant birth outcomes.

## 2. Materials and Methods

### 2.1. Sample

Pregnant women were recruited from prenatal clinics that served the study communities in the Western Cape Province of South Africa. Women were invited to participate if they were between 5–36 weeks gestation and reported: (a) alcohol use during the past seven days (*n* = 14); (b) alcohol use during the past 30 to 90 days (*n* = 22); or (c) abstention over the past 90 days (*n* = 16). Women were predominately of mixed-race “Coloured” ancestry. Women provided a fasting bloodspot sample from finger pricks. The average week of gestation at the time of bloodspot collection was 18.5 (±6.5). Following the birth, the infant’s length, weight, and occipitofrontal (head) circumference (OFC) were measured by the attending physician or nurse and measurements were recorded on the infant’s permanent clinic card. All participants provided written informed consent. Samples were stored at −80 °C until processed. All procedures were approved by the Ethics Committee of Stellenbosch University, Faculty of Medicine and Health Sciences, and the University of North Carolina.

### 2.2. Metabolomic Analysis

An untargeted metabolite analysis of the bloodspot samples was performed by Metabolon, Inc. (Durham, NC), using a proprietary process as detailed in Saini et al. [34]. In summary, samples were analyzed using reverse phase/ultra-high-performance liquid chromatography-mass spectrometry (UPLC-MS)/MS with ion mode electrospray ionization (both positive and negative) [35]. Samples were extracted, dried, and reconstituted with compatible solvents for each analytic technique. Two aliquots were analyzed with acidic positive ion conditions—one was chromatographically optimized for hydrophilic compounds and the other chromatographically optimized for hydrophobic compounds. A third and a fourth aliquot were analyzed with basic negative ion optimization. The order of sample analysis was randomized and regularly interspersed with known quantitative and qualitative standards. Peaks were identified based on retention index, mass, and chromatographic data. Area-under-the-curve (AUC) was used to quantify the peaks [36].

### 2.3. Data Analysis

For the metabolites, the assumptions of normality and equal variance were not met when assessed using Levene’s and Shapiro–Wilks tests. Non-parametric Mann-Whitney U-tests (Wilcoxon Rank sum test) were subsequently carried out. Metabolites that were below the detection limit for more than half of the participants were excluded from the analysis. If fewer than half were missing, the missing values were imputed using the minimum value for the specific metabolite. To correct for multiple comparisons, *p*-values were adjusted using Benjamini–Hochberg False Discovery Rate (FDR) and are presented as q-values. Fold change (FC) was calculated as the mean of the exposure group relative to mean of the reference group. A q-value of 0.05 was considered significant. Metabolites approaching significance were noted when 0.05 ≤ q ≤ 0.10. Principal component analysis (PCA), partial least square discriminant analysis (PLS-DA), k-means clustering, and data visualization were performed in R (version 4.1) [37] with the MetaboAnalystR package (version 3.2) [38]. PCA reduces the dataset’s dimensionality through linear transformation to reduce and identify sources of variance in the dataset. The PLS-DA similarly optimizes group separation but by maximizing the covariance between the observed variables (e.g., metabolites) and class membership. In k-means clustering, allocation of individual-level data to a fixed number of groups (k) is achieved by partition groups such that in-group sum of squares is minimized. Because ethyl alpha-glucopyranoside and ethyl beta-glucopyranoside were expected to drive the difference between the two groups, both were excluded from the PCA, PLS-DA, and k-means clustering. Scores from the PLS-DA variable importance projection (VIP) were used to determine the contribution of each metabolite to the model.

To identify metabolites associated with infant birth outcomes, ortho-PLSDA (oPLS-DA) was performed using the ropls package (version 1.28.2) [39] with scaled z-score metabolites as the predictors. To identify the influential metabolites associated with infant birth outcomes, the top 30 VIP scores were extracted for the first orthogonal component. Pearson’s correlation analyses with the top 30 VIP scores and infant birth measures were carried out with the rstatix package (version 0.7.0) [40] and plotted with ggpubr (version 0.4.0) [41]. Two mother/infant dyads were missing birth outcome measures and were excluded from the oPLS-DA.

## 3. Results

### 3.1. Maternal Characteristics

Table 1 displays the maternal characteristics. Approximately 70% of the participants reported consuming alcohol before pregnancy and in the 1st trimester of pregnancy. Among those who reported alcohol consumption, women consumed, on average, 6.1 drinks per drinking day (DDD) with a mean of 1.8 days per week prior to pregnancy. During the 1st trimester, women who consumed alcohol reported, on average, 5.8 DDD on 1.8 days per week. Among those who drank in the previous seven days, women reported 4.0 DDD on 1.9 days per week. Women who used alcohol recently (in the previous week) were, on average, older and had higher gravidity and parity than women who did not drink recently. Tobacco use during pregnancy was reported by many mothers (53.8%), and none reported using other drugs during pregnancy.

### 3.2. Metabolite Profiles

The untargeted metabolite analysis identified 860 biochemicals of which 772 were known compounds and 88 were unidentified biochemicals. A total of 78 metabolites were below the detection limit for more than half of the participants and were excluded from further analysis, leaving 782 biochemicals of which 702 were known and 80 were unknown. There was no clear differentiation based on sex of the offspring (Figure S1); therefore, all mothers were analyzed together. Because metabolites are dynamic and can fluctuate based on environmental factors (e.g., dietary intake, nutrient stores, and toxic agents), women were classified as alcohol consuming if they reported alcohol consumption in the seven days prior to the bloodspot collection.

In total, 44 of the 782 metabolites (5.6%) significantly differentiated women who consumed alcohol in the previous 7 days (“Alc”) from women who abstained in the previous 7 days (“Con”) (q ≤ 0.05 with Mann–Whitney U-test with Benjamini–Hochberg correction). A total of 12 of the 44 significant metabolites (27.3%) were enriched by alcohol consumption, while 32 (72.7%) were reduced. An additional 45 metabolites had q-values between 0.05 and 0.10; nine were enriched, and 36 were reduced by alcohol consumption (Table 2). Given the quantity of drinks per drinking day reported by women who consumed alcohol in the previous 7 days, a 9.4-fold increase in ethyl beta-glucopyranoside over women who did not report drinking in the previous 7 days was expected. A 2.8-fold increase in cotinine, a nicotine metabolite, was also expected given that approximately 85% of mothers who consumed alcohol reported tobacco use.

Alcohol consumption significantly increased the abundance of seven acyl carnitine derivatives involving both short- and medium-chain fatty acids (Table 2). Two alpha-keto-catabolites (isovalerylcarnitine and beta-hydroxyisovaleroylcarnitine) of the branch chain amino acid leucine were significantly enriched. In contrast, two tryptophan amino acid catabolites, indole-3-carboxylate (0.81-fold) and kynurenate (0.84-fold), and the gamma-amino acid 4-guanidinobutanoate (0.85-fold), were reduced in the alcohol-consuming group.

Although sphingomyelins represent only 3.5% of the metabolites analyzed (*n* = 27), alcohol consumption significantly (q ≤ 0.05) reduced the abundance of 14 sphingomyelins (51.9% of all sphingomyelins detected), with fold-changes ranging from 0.84 to 0.57 (Table 2). Three additional sphingomyelin reductions approached significance (q ≤ 0.10). Several other lipids were also reduced, including several lyso-phospholipids, free fatty acids, and two oxidized lipids (9,10-DiHOME and 15-HETE). Interestingly, the abundance of cholesterol and pregnenolone-derived steroids was reduced, whereas several androsterone metabolites were significantly elevated by alcohol consumption. Cortisol was almost twice as high in the alcohol-consuming group. Phytochemicals, microbial productions, and other metabolites were also generally reduced by alcohol consumption.

**Figure 1:**
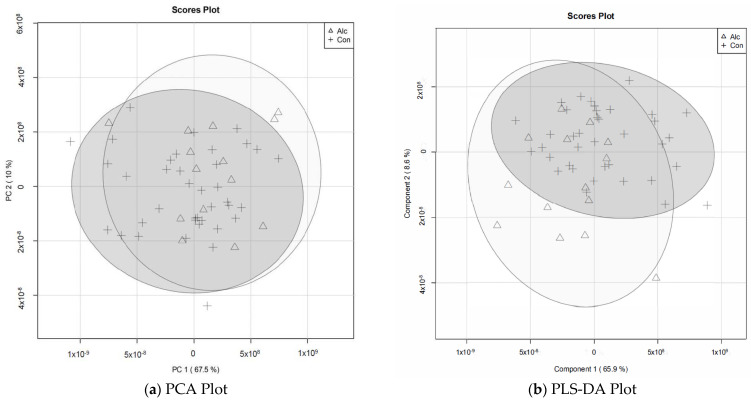

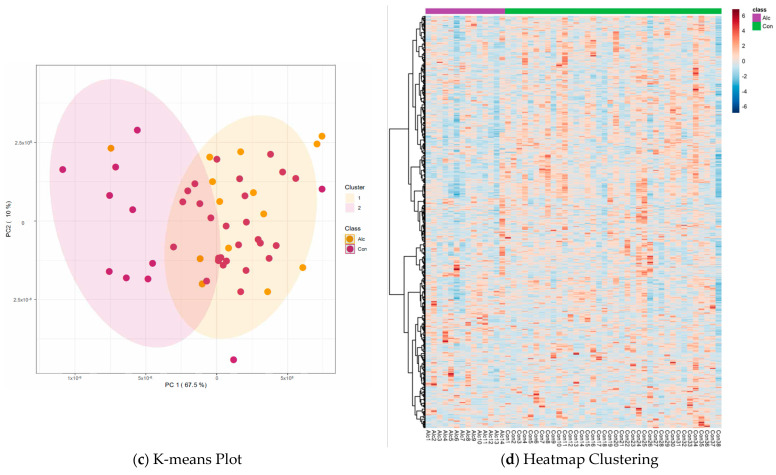

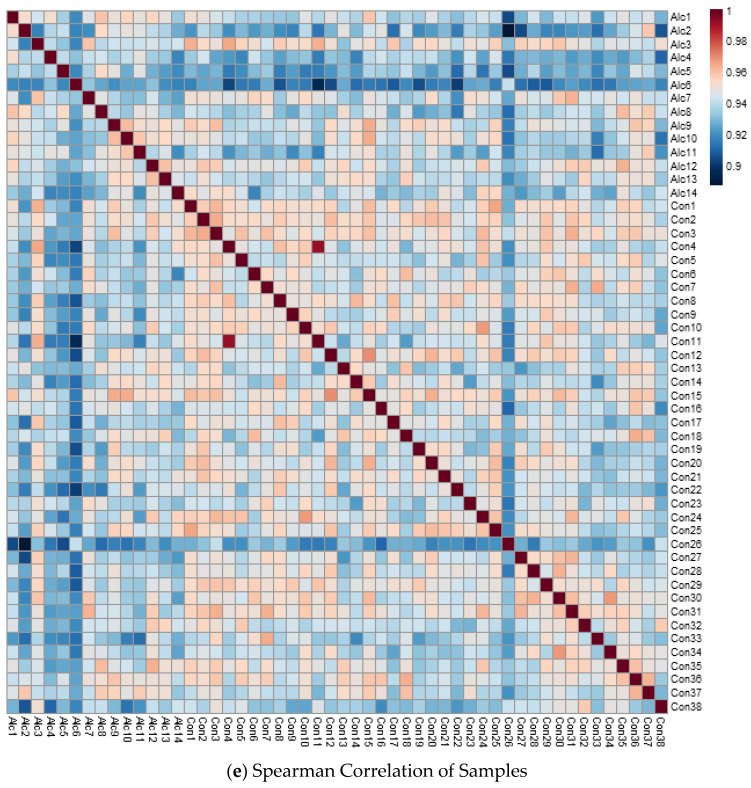
Multivariate analyses of metabolite profiles among pregnant women who consumed alcohol in the previous seven days and abstainers (*n* = 52). The analyses (**a**) Principal Component Analysis, (**b**) partial least squares—discriminate analysis (PLS-DA), (**c**) k-means clustering, (**d**) heatmap clustering, (**e**) Spearman correlation of samples were unable to separate women who consumed alcohol from abstainers. In the heatmap, a red to blue gradient represents the high, unchanged, and low abundance. In the correlation of samples, a red to blue gradient represent the positive, unchanged, and negative correlations. Alc denotes drinking within the past 7 days. Con denotes did not drink within the past 7 days.

**Figure 2:**
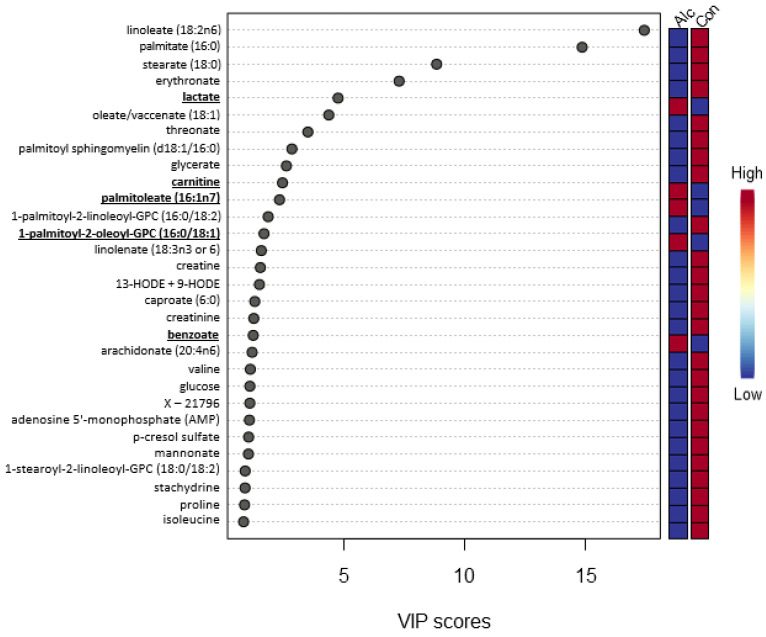
Variable importance projection (VIP) score plot with the top 30 metabolites that contribute to the separation of the metabolite profiles in Component 1 in the PLS-DA among women who consumed alcohol and abstainers. **Bold** metabolites were significantly enriched in those consuming alcohol. Red boxes denote an increased mean metabolite abundance. Blue boxes denote a decreased mean metabolite abundance. Alc denotes drinking within the past 7 days. Con denotes did not drink within the past 7 days.

To distinguish metabolite profiles of women who reported alcohol consumption in the previous 7 days from women who did not, multivariate analyses were undertaken. Although there was virtually no separation between the groups, Principal Component (PC) 1 explained 67.5% of the variance and PC2 explained 10.0% of the variance (Figure 1a). PC1 was driven primarily by medium-chain free fatty acids: palmitate, linoleate, oleate/vaccenate, and stearate (Table S1). PC2 was driven by erythronate, oleate/vaccenate, stearate, and glycerate (Table S2). PCA biplots are provided in the Appendix (Figures S2 and S3). As revealed by the scree plot (Figure S4), no additional separation was achieved in PC3 (5.7%), and thus further PCs were not explored. Likewise, the PLS-DA (Figure 1b), k-means clustering (Figure 1c), heatmap cluster (Figure 1d), and Spearman correlations (Figure 1e) were unable to clearly differentiate between women who consumed alcohol in previous 7 days and those who did not. Given the limited separation between groups, we evaluated the variable importance projection (VIP) scores for the top 30 metabolites which contributed to the variance in the PLS-DA to gain additional insight into their relationships (Figure 2). Most of the metabolites that contributed to the variance in the PLS-DA (25 of the 30, 83.3%) were reduced among women who consumed alcohol. Among the greatest influences were medium-chain free fatty acids (palmitate, stearate, oleate, and linoleate), oxidized sugar derivatives (glycerate, erythronate, and threonate), which were all decreased, and lactate which was elevated. Because only eight metabolites with significantly altered abundances were annotated in KEGG, a formal pathway analysis to obtain metabolic insights was not performed.

### 3.3. Maternal Metabolite Profiles Associated with Infant Birth Measurements

**Figure 3:**
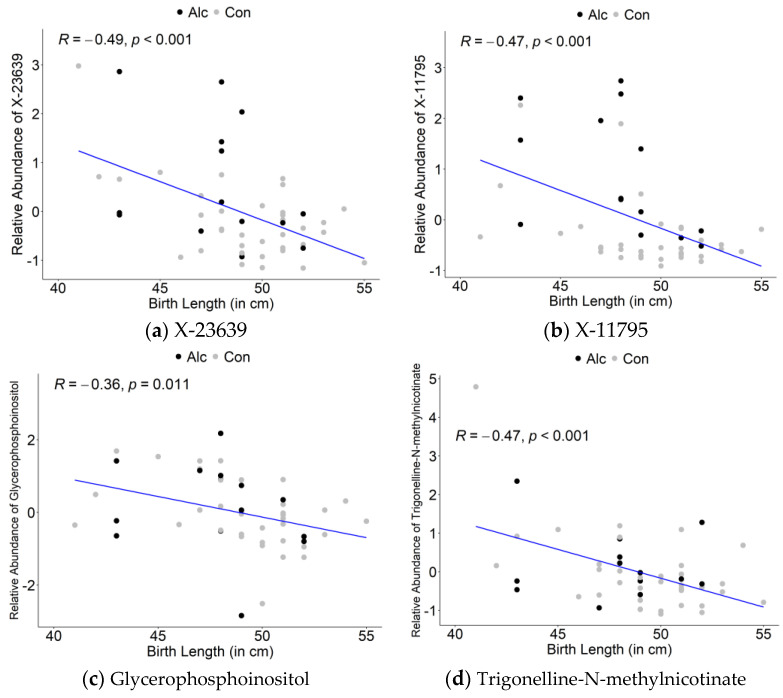
Correlation plots of top metabolites identified in oPLSDA with infant birth length: (**a**) X-23639; (**b**) X-11795; (**c**) Glycerophosphoinositol; (**d**) Trigonelline-N-methylnicotinate. Alc denotes drinking within the past 7 days.

We used oPLS-DA to explore associations between maternal metabolites and the infant birth outcomes of length, weight, and occipitofrontal (head) circumference (OFC). This revealed that the maternal metabolites could be separated on the basis of these infant outcomes (Figure S5). With respect to infant birth length, of the top 30 maternal metabolites in Component 1 of the oPLS-DA, half (50%) were lipid-related and 8 (26.7%) were unidentified compounds; other metabolites included glutamate, myo-inositol, glycerol-3-phosphate, and the coffee-enriched niacin trigonelline (Table 3). The majority (76.7%) of these maternal metabolites were negatively associated with infant birth length. Correlation plots for the top metabolites associated with infant birth length are shown in Figure 3 and all were negatively correlated.

**Figure 4:**
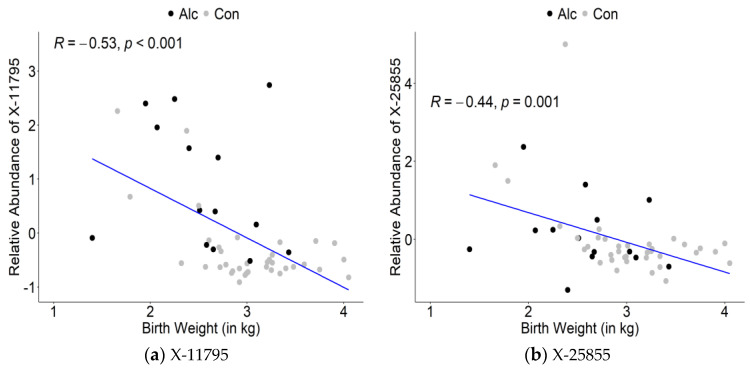

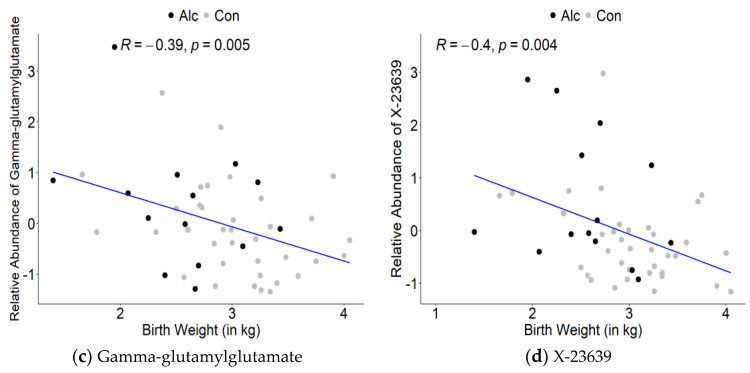
Correlation plots of top metabolites identified in oPLSDA with infant birth weight: (**a**) X-11795; (**b**) X-25855; (**c**) Gamma-glutamylglutamate; (**d**) X-23639. Alc denotes drinking within the past 7 days. Con denotes did not drink within the past 7 days.

Con denotes did not drink within the past 7 days.Similarly, the top 30 maternal metabolites in the oPLS-DA that were correlated with infant birth weight were largely lipid-related (13, 43.3%) or unidentified compounds (8, 26.7%) (Table 4). Additional associations were found for glycerol-3-phosphate, myo-inositol, glutamate, glycine, sphingosine-1-phosphate, and cotinine. Again, nearly all (26 out of 30, 86.7%) were negatively correlated with infant birth weight with correlation values ranging from r = −0.53 to −0.22. Figure 4 shows the correlation plots for the top metabolites (X-11795, X-25855, gamma-glutamylglutamate, X-23639) which were all negatively associated with infant birth weight.

**Figure 5:**
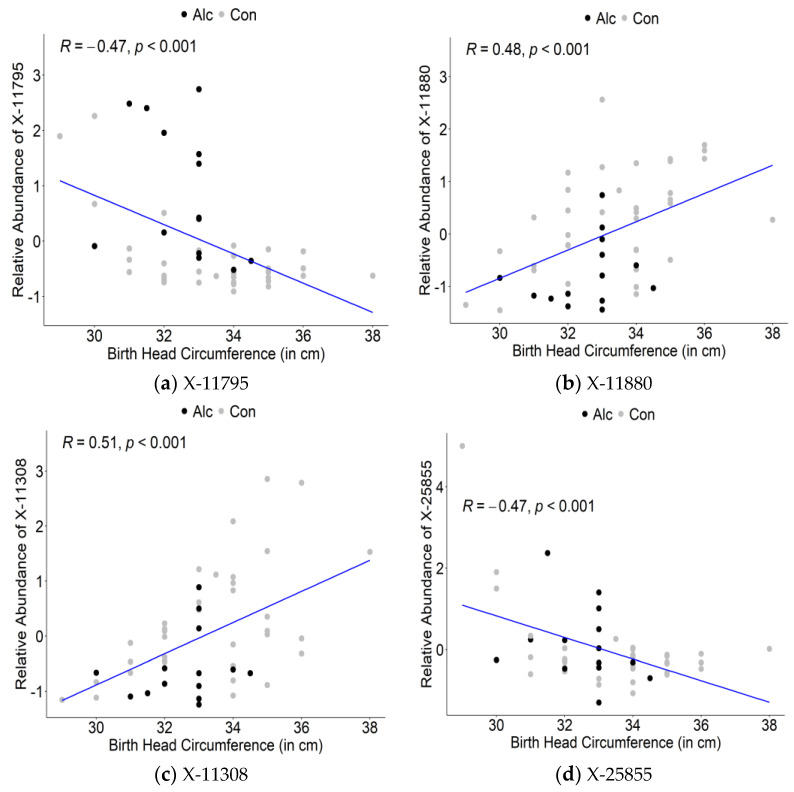
Correlation plots of top metabolites identified in oPLSDA with infant birth head circumference: (**a**) X-11795; (**b**) X-11880; (**c**) X-11308; (**d**) X-25855. Alc denotes drinking within the past 7 days. Con denotes did not drink within the past 7 days.

The top 30 maternal metabolites associated with infant OFC were again largely lipid-related (11, 36.7%), unidentified compounds (9, 30.0%), and again included cotinine, guanosine, and glycine but also included several sphingomyelins and xenobiotics (Table 5). Although 17 of the metabolites were negatively associated with infant birth OFC, 13 metabolites were positively associated with infant birth OFC, including the maternal sphingomyelins (correlation coefficients ranging from 0.42 to 0.35). Among the top metabolites associated with infant OFC, two (X-11880 and X-11308) were positively associated with OFC and two (X-11795 and X-25855) were negatively associated (Figure 5).

**Figure 6:**
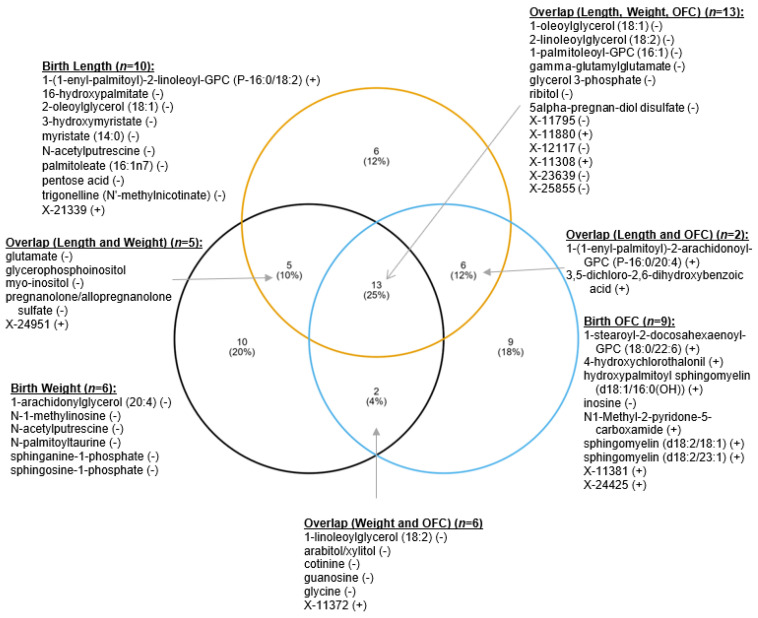
Overlap of metabolites associated with infant birth length, weight, and head circumference (OFC). (-) negative association with infant measure. (+) positive association with infant measure.

Figure 6 depicts the overlap in the metabolites from the oPLS-DA which were associated with the three infant birth measurements. In total, 13 metabolites were significantly associated with all 3 infant birth measurements. X-11795, which has previously been associated with wine consumption [42], was negatively correlated with infant birth length, weight, and OFC. The monoacylglycerols for oleate and linolenate, lysophosphatidylcholine, 5-alpha-preganan-diol disulfate and glycerol 3-phosphate were also negatively associated with all three infant birth measurements. For two sugar alcohols, ribitol was negatively associated with length, weight, and OFC, while arabitol, an intermediate of the pentose-phosphate pathway, was negatively associated with infant birth weight and OFC. Only two metabolites (X-11800 and X-11308) were positively associated with all three infant birth measurements, and two others (X-11372 and X-24951) were positively associated with two of the infant outcomes. Cotinine was significantly and negatively associated with infant weight and OFC.

## 4. Discussion

The key finding from this study is that maternal alcohol consumption altered the blood metabolomic profile, and some metabolites were significantly associated with infant birth measurements. Metabolomic changes included: an increase in multiple acyl carnitine and amino acid carnitine catabolites, suggesting an increase in protein and fatty acid degradation and a reduction in the specialized lipids, sphingomyelins, and their precursors. The consistency of the metabolite associations which emerged are remarkable given that these samples were taken at various times during pregnancy and the women had freedom in dietary choice.

### 4.1. Energy Utilization Alterations: Amino Acid Catabolites and Acyl Carnitines

Glucose, derived from maternal plasma pools, is the primary energy source for the fetus. If maternal glucose plasma pools are insufficient for meeting the demands for herself, the placenta, and the fetus, alternative energy sources will be utilized. Our prior preclinical studies identified a reduction of maternal hepatic glucose and increased abundance of amino acid catabolites, findings that suggest the increase in amino acid catabolites may be an attempt to meet the increased demand for energy during pregnancy [11]. We found similar associations between alcohol consumption and catabolites of the essential branched chain amino acid leucine. Their enhancement suggests that energy sources other than glucose were used to meet the energy demands of the mother and fetus.

Fatty acid oxidation intermediates (acyl carnitines) were also significantly elevated by alcohol consumption. β-oxidation supplies acetyl-CoA to the tricarboxylic acid (TCA) cycle to produce adenosine triphosphate (ATP) for energy from non-glucose origins. This accumulation of acyl carnitines could reflect the increased lipolysis that accompanies alcohol exposure [43] and could suggest an inefficient oxidation of acyl-CoA species. Supporting this is the reduction in the key TCA intermediate citrate, the first product in obtaining energy from lipid- and glucose-derived acetyl-CoA. Importantly, in this study and our prior preclinical study, we found that glycolytic intermediates and amino acid metabolites—in maternal liver for mouse [11] and bloodspots for human—were consistently correlated with measures of fetal growth. Although glucose itself did not emerge in the oPLS-DA, collectively, these findings suggest that maternal glucose was insufficient or not utilized appropriately to meet the increased demands during pregnancy. Given their negative correlation with fetal outcomes, these associations imply that fetal energy needs may not have been met. Alcohol’s ability to compete with nutrients via shared metabolic pathways and to disrupt placental blood flow and nutrient transportation may further place a fetus at risk [44,45,46].

### 4.2. Lipid-Derived Alterations

In addition to their role in supplying and storing energy, lipids also have non-energy roles as bioactives that play regulatory and hormonal roles. Notably, one class of lipids, sphingomyelins, had fourteen analogs significantly reduced in women who consumed alcohol. Sphingomyelins are comprised of ceramide (sphingosine plus a fatty acid) and a polar head group (often phosphocholine) and play important roles in membrane dynamics, including their formation (e.g., myelin sheath surrounding nerve cell axons), stabilization (e.g., lipid raft formation), cell adhesion and migration, and cell signaling (e.g., apoptosis) [47,48]. The broad reductions in sphingomyelins suggest that alcohol altered one or more key steps in their synthesis or turnover. Whether these differences in bloodspots reflect an altered cell membrane composition (predominantly red blood cells) or plasma very low-density lipids (VLDL) could not be determined. As significant components of brain myelin, these reductions might inform the altered white matter content and deficits in information processing and cognitive abilities that typify those with FASD [49,50].

Alcohol is known to increase cortisol levels and stimulate the hypothalamic–pituitary–adrenal (HPA) axis leading to an increase in adrenal androgen production [51,52]. Sulfated steroids, such as pregnenetriol disulfate and androstenediol (3beta,17beta) disulfate (1), can regulate synaptic transmission via changes in postsynaptic neurotransmitter receptors, and alterations of sulfation steroids have been implicated as one mechanism contributing to FASD [53]. In this context, it may be notable that we identified elevations in androsterones and reductions in their upstream pregnenolones and cholesterol in the women who recently consumed alcohol.

### 4.3. Other Metabolic Alternations: Phytochemicals, Microbial Products, and Other Metabolites

The reduction in phytochemicals among women who recently consumed alcohol contrasts with our prior findings in a preclinical model of PAE in which the alcohol-exposed maternal plasma was enriched in phytochemicals derived from microbial fermentation of dietary fibers [12]. However, that model used a fixed-nutrient diet, and we speculate that the reductions seen here represent a reduction in phytochemical-rich foods in the diet of the women who consumed alcohol; phytochemicals are not stored and typically have a half-life of hours to a few days. Alcohol consumption is also known to alter the microbiome [54]. Offspring exposed to PAE have an altered bacterial diversity and community structure compared with unexposed offspring [55], suggesting that the maternal microbiome is likely to be impacted. However, such maternal effects represent a gap in this research.

### 4.4. Maternal Metabolites Influence Infant Birth Outcomes

Importantly, our correlation analyses suggest that these changes in maternal metabolites associate with measures of infant development. We identified a common set of maternal metabolites that were consistently associated with infant birth length, weight, and OFC. Nearly all these associations were negative, implying they represent processes having adverse consequences for fetal development. These included two monoacylglycerols, a lysolecithin, and glycerol-3-phosphate, and they may represent the action of lipases on plasma VLDL, as red cells lack oxidative phosphorylation.

Sphingomyelins were positively associated with infant OFC, a surrogate for brain weight, whereas their precursors, sphingosine- and sphinganine-1-phosphate, were negatively correlated with growth, changes that suggest an impairment of sphingomyelin synthesis. Their synthesis depends upon adequate choline and the one-carbon pool and could represent one of several mechanisms by which supplemental choline benefits brain development [45,56]. Sphingomyelins comprise approximately 35% of the lipids in myelin [57], and brain imaging studies of children with FASD identify subtle changes in white matter that are associated with an individual’s neurocognitive impairments [50,58,59]. The reduction in sphingomyelins observed here may partially explain these white matter reductions.

Multiple unidentified compounds differentiated the maternal groups and were also associated with infant outcomes. The compound X-11795 is associated with wine consumption [42]; it was elevated among women who consumed alcohol in the previous 7 days and was negatively associated with infant birth outcomes, consistent with the teratogenic effects of PAE. Compound X-12117 was negatively correlated with all infant birth measurements, and has been previously linked to adverse health outcomes including renal disease, cardiovascular disease, and all-cause mortality [60]. Conversely, four unknown compounds were positively associated with at least two infant birth outcomes. Fried foods are associated with X-11372 [61], which was positively associated with infant weight and OFC. In this population where undernutrition is common [24,25], X-11372 increase could indicate greater consumption of calorically-dense foods and may be protective for infant growth. Compound X-11800, which correlates with vegetable consumption [62], and compound X-11308, which is likely a polyunsaturated fatty acid [63], were both positively associated with infant length, weight, and OFC. A diverse diet rich in polyunsaturated fats and vegetables is advantageous for fetal development.

### 4.5. Limitations

This study demonstrated altered metabolite profiles among women who consumed alcohol relative to abstainers; however, there were limitations. First, this is a relatively small sample in which a single timepoint was analyzed; moreover, the gestational age for the bloodspot collection varied by woman. The women who consumed alcohol were older and had higher gravidity/parity, and the latter may negatively affect maternal nutrient stores prior to the index pregnancy. Second, unsupervised data modeling demonstrated little separation between the two groups. Nevertheless, despite these limitations, our untargeted analysis still identified a unique metabolic profile among the women who recently consumed alcohol.

## 5. Conclusions

In summary, we used an untargeted metabolomics approach to identify a metabolic profile that characterized the bloodspots from women who consumed alcohol in the prior seven days and distinguished them from those who had not consumed alcohol during that period. This profile emerged even though the women had different dietary patterns and were sampled at times of pregnancy ranging from 5 to 36 weeks of gestation. These metabolite differences suggest an increased catabolism of amino acids and fatty acids, reduced sphingomyelins, and elevated androsterone production in those who recently consumed alcohol. These increases in amino acid and fatty acid catabolism are mirrored in our preclinical mouse model of alcohol-exposed pregnancy, supporting that model’s relevance for these studies. A common set of metabolites emerged that correlated with newborn length, weight, and OFC. Taken together, a unique metabolic pattern differentiated women who consumed alcohol from abstainers and these findings validate untargeted metabolomics to generate mechanistic insights into growth impairments and FASD.

## Figures and Tables

**Table 1:** Maternal Demographic and Alcohol Consumption History.

	All Women ^1^ (*n* = 52)		Alcohol-Exposed (Past 7 Days) (*n* = 14)		No Alcohol Consumption in Past 7 Days (*n* = 38)		*p* ^1^
**Demographics**	Mean	(SD)	Mean	(SD)	Mean	(SD)	
Height (in cm)	156.7	(7.0)	156.9	(6.1)	156.6	(7.3)	0.890
Weight (in kg)	65.7	(19.4)	62.4	(18.8)	67.0	(19.6)	0.450
Body Mass Index (BMI)	26.8	(7.9)	25.4	(7.8)	27.4	(8.0)	0.438
OFC (in cm)	53.8	(1.9)	53.5	(1.8)	53.8	(2.0)	0.696
Left Upper Arm (in cm)	26.5	(5.4)	24.9	(3.8)	27.1	(5.8)	0.197
Age at Interview	27.2	(6.5)	30.3	(5.5)	26.1	(6.5)	0.036
Gravidity	2.8	(1.3)	3.5	(1.5)	2.5	(1.2)	0.013
Parity	1.6	(1.2)	2.3	(1.3)	1.4	(1.1)	0.015
**Self-Reported Alcohol & Other Drugs**							
Drank Before Pregnancy (% Yes)	71.2		100.0		60.5		0.005
Avg. DDD—before pregnancy ^2^	6.1	(2.8)	6.1	(2.8)	6.1	(2.9)	0.877
Number of drinking days—before pregnancy ^2^	1.8	(0.9)	2.2	(1.1)	1.6	(0.8)	0.061
Drank in 1st trimester (% Yes)	69.2		100.0		57.9		0.004
Avg. DDD—in 1st trimester ^2^	5.8	(2.9)	5.6	(3.0)	2.9	(3.0)	0.799
Number of drinking days—in 1st trimester ^2^	1.8	(1.0)	2.2	(1.2)	1.6	(0.8)	0.067
Drank recently (in the previous 7 days) during pregnancy (% Yes)	26.9		100.0		0.0		<0.001
Avg. DDD—previous 7 days ^2^	4.0	(2.1)	4.0	(2.1)	--	--	--
Number of drinking days—previous 7 days ^2^	1.9	(0.9)	1.9	(0.9)	--	--	--
Used tobacco during pregnancy (% Yes)	53.8		85.7		57.9		0.005
Used other drugs during pregnancy (% Yes)	0.0		0.0		0.0		--
**Alcohol Biomarker: Relative Abundance**							
Ethyl alpha glucopyranoside	130,957.1 (248,451.9)		283,790.6 (429,289.4)		68,025.62 (36,433.28)		0.245
Ethyl beta glucopyranoside ^3^	288,558.4 (638,093.5)		574,988.8 (943,709.4)		92,579.74 (87,786.57)		0.020

^1^. The “all women” column was excluded from the statistical test. ^2^. Among those who reported alcohol consumption in that time period. ^3^. Alcohol-exposed (past 7 days) *n* = 13; No alcohol consumption in past 7 days *n* = 19. DDD: Drinks per Drinking Day; OFC: Occipital frontal (head) circumference.

**Table 2:** Relative Abundance of Metabolites Which Significantly Differentiated Women Who Consumed Alcohol in Previous 7 Days (*n* = 14) from Women who Abstained (*n* = 38).

Biochemical Name	Relative Abundance Mean		Fold Change ^1^	log_2_(FC)	FDR ^2^
	Alc	Con			
**Acyl Carnitines**					
carnitine	118,352,430	96,072,262	1.23	0.30	0.07914
acetylcarnitine (C2)	9,728,562	5,937,530	1.64	0.71	0.084043
isobutyrylcarnitine (C4)	900,579	594,692	1.51	**0.60**	0.037839
(R)-3-hydroxybutyrylcarnitine (C4)	91,719	50,728	1.81	**0.85**	0.021685
(S)-3-hydroxybutyrylcarnitine (C4)	82,182	46,777	1.76	**0.81**	0.015661
2-methylbutyrylcarnitine (C5)	174,872	94,909	1.84	**0.88**	0.035527
isovalerylcarnitine (C5)	624,704	450,542	1.39	0.47	0.057764
beta-hydroxyisovaleroylcarnitine	1,902,421	1,294,463	1.47	**0.56**	0.025134
hexanoylcarnitine (C6)	263,025	185,087	1.42	0.51	0.057764
myristoleoylcarnitine (C14:1)	238,415	144,643	1.65	**0.72**	0.021757
palmitoleoylcarnitine (C16:1)	834,256	499,204	1.67	**0.74**	0.015661
**Amino Acid Catabolites**					
indole-3-carboxylate	513,556	630,299	0.81	−0.30	0.057764
kynurenate	197,290	235,324	0.84	−0.25	0.082970
4-guanidinobutanoate	7,039,465	8,259,819	0.85	−0.23	0.085952
**Sphingomyelins**					
sphingomyelin (d17:1/16:0, d18:1/15:0, d16:1/17:0)	4,083,609	5,660,430	0.72	**−0.47**	0.02645
sphingomyelin (d18:1/17:0, d17:1/18:0, d19:1/16:0)	2,344,136	3,179,638	0.74	**−0.44**	0.02501
sphingomyelin (d18:1/19:0, d19:1/18:0)	547,267	786,114	0.70	**−0.52**	0.04540
sphingomyelin (d18:1/20:0, d16:1/22:0)	7,422,894	8,847,552	0.84	**−0.25**	0.04937
sphingomyelin (d18:1/20:1, d18:2/20:0)	4,128,434	5,413,863	0.76	**−0.39**	0.02176
sphingomyelin (d18:1/21:0, d17:1/22:0, d16:1/23:0)	1,086,342	1,557,715	0.70	**−0.52**	0.02501
sphingomyelin (d18:1/22:1, d18:2/22:0, d16:1/24:1)	12,137,149	14,773,882	0.82	−0.28	0.05163
sphingomyelin (d18:1/22:2, d18:2/22:1, d16:1/24:2)	1,283,423	1,626,160	0.79	**−0.34**	0.021757
sphingomyelin (d18:2/16:0, d18:1/16:1)	13,534,533	17,191,969	0.79	−0.35	0.062328
sphingomyelin (d18:2/18:1)	223,567	354,266	0.63	**−0.66**	0.049366
sphingomyelin (d18:2/21:0, d16:2/23:0)	248,474	439,680	0.57	**−0.82**	0.021685
sphingomyelin (d18:2/23:0, d18:1/23:1, d17:1/24:1)	3,435,091	4,537,665	0.76	**−0.40**	0.021757
sphingomyelin (d18:2/23:1)	391,078	551,118	0.71	**−0.49**	0.026446
sphingomyelin (d18:2/24:2)	1,557,823	2,113,087	0.74	**−0.44**	0.021757
tricosanoyl sphingomyelin (d18:1/23:0)	2,795,976	3,461,568	0.81	**−0.31**	0.025013
hydroxypalmitoyl sphingomyelin (d18:1/16:0(OH))	572,980	760,311	0.75	**−0.41**	0.021757
palmitoyl dihydrosphingomyelin (d18:0/16:0)	7,312,117	8,336,650	0.88	−0.19	0.084043
**Steroids**					
cholesterol	10,923,644	12,495,807	0.87	**−0.19**	0.021757
cortisol	145,659	76,815	1.90	0.92	0.084043
5alpha-androstan-3beta,17beta-diol disulfate	159,481	75,476	2.11	**1.08**	0.025134
5alpha-pregnan-3beta,20alpha-diol monosulfate (2)	670,377	1,170,702	0.57	−0.80	0.059394
pregnenediol sulfate (C21H34O5S)	245,883	411,828	0.60	−0.74	0.057764
pregnenetriol sulfate	56,527	90,669	0.62	−0.68	0.085952
androstenediol (3beta,17beta) disulfate (1)	3,485,474	1,000,067	3.49	**1.80**	0.046245
**Specialized Lipids**					
(2 or 3)-decenoate (10:1n7 or n8)	111,491	140,550	0.79	−0.33	0.084043
pentadecanoate (15:0)	40,396,503	480,519,34	0.84	−0.25	0.059394
N-palmitoylserine	245,875	308,316	0.80	−0.33	0.059394
palmitoyl ethanolamide	175,718,599	201,504,407	0.87	**−0.20**	0.021757
linoleoyl ethanolamide	52,234	95,443	0.55	**−0.87**	0.037839
glycochenodeoxycholate 3-sulfate	218,818	87,130	2.51	1.33	0.084043
1-linoleoyl-GPA (18:2)	396,446	581,860	0.68	−0.55	0.076617
1-linoleoyl-GPC (18:2)	5,782,388	7,670,952	0.75	**−0.41**	0.025013
1-oleoyl-GPS (18:1)	825,394	1,271,934	0.65	**−0.62**	0.049366
1-(1-enyl-palmitoyl)-2-linoleoyl-GPC (P-16:0/18:2)	1,166,995	1,594,375	0.73	**−0.45**	0.026446
2-hydroxyheptanoate	3,152,157	3,776,354	0.83	−0.26	0.056498
alpha-hydroxycaproate	413,348	494,072	0.84	−0.26	0.096824
9,10-DiHOME	1,241,335	1,655,580	0.75	−0.42	0.057764
15-HETE	2,851,244	3,968,058	0.72	−0.48	0.098106
2-hydroxysebacate	323,393	389,143	0.83	−0.27	0.098106
3-carboxy-4-methyl-5-pentyl-2-furanpropionate (3-CMPFP)	338,493	578,390	0.59	**−0.78**	0.046245
eicosenedioate (C20:1-DC)	109,938	188,788	0.58	−0.78	0.057764
2-aminoheptanoate	134,956	209,995	0.64	**−0.64**	0.021685
**Phytochemicals / Microbial Products**					
p-hydroxybenzaldehyde	7,560,374	9,289,475	0.81	**−0.30**	0.037236
4-hydroxybenzoate	1,444,142	1,837,542	0.79	−0.35	0.057764
2-oxindole-3-acetate	51,328	66,566	0.77	−0.38	0.090168
3-formylindole	6,900,965	8,152,590	0.85	**−0.24**	0.021757
3-phenylpropionate (hydrocinnamate)	53,859	95,887	0.56	−0.83	0.079140
3-methyl catechol sulfate (1)	188,585	110,852	1.70	0.77	0.070938
o-cresol sulfate	93,537	55,448	1.69	0.75	0.057764
piperine	25,012	251,263	0.10	**−3.33**	0.025349
pyrraline	24,706	36,774	0.67	−0.57	0.059394
**Other Metabolites**					
citrate	14,310,699	17,171,422	0.83	−0.26	0.084043
oxalate (ethanedioate)	15,630,699	18,204,085	0.86	−0.22	0.059394
3-aminoisobutyrate	538,044	355,236	1.51	0.60	0.084043
6-phosphogluconate	502,951	580,785	0.87	−0.21	0.096824
acetylphosphate	10,654,105	15,392,171	0.69	−0.53	0.096824
phenylacetylglutamine	645,354	1,128,644	0.57	−0.81	0.098106
Fibrinopeptide B (1–13)	773,890	1,038,347	0.75	−0.42	0.054009
**Others**					
cotinine	1,738,769	620,542	2.80	**1.49**	0.025013
ethyl beta-glucopyranoside	535,423	56,820	9.42	**3.24**	0.021685
(2-butoxyethoxy)acetic acid	2,144,519	2,598,232	0.83	**−0.28**	0.026446
N-formylanthranilic acid	2,005,367	2,407,944	0.83	−0.26	0.059394
4-hydroxychlorothalonil	148,112	244,884	0.60	−0.73	0.084043
succinimide	131,745	208,990	0.63	−0.67	0.096824
X-11308	346,764	541,090	0.64	−0.64	0.057764
X-11372	1,035,122	1,735,238	0.60	**−0.75**	0.021757
X-11795	724,612	291,990	2.48	**1.31**	0.015661
X-11880	491,137	848,065	0.58	**−0.79**	0.021757
X-16935	33,165	54,359	0.61	−0.71	0.070938
X-18059	2,566,792	3,184,326	0.81	**−0.31**	0.025013
X-21286	420,340	556,471	0.76	**−0.40**	0.025013
X-21628	384,031	605,092	0.63	**−0.66**	0.015909
X-23481	646,716	761,238	0.85	−0.24	0.098106
X-23482	3,108,264	4,128,471	0.75	−0.41	0.085952
X-24951	127,673	209,085	0.61	**−0.71**	0.033020

^1^ Relative Abundance Mean_Alc_ divided by Relative Abundance Mean_Con_; ^2^ Mann–Whitney U-test, followed by Benjamini–Hochberg False Discovery Rate (FDR) adjustment; **Bold** log_2_(FC) denotes significance (q < 0.05). Relative abundance are normalized in terms of raw area counts and represent raw intensity

**Table 3:** Top 30 metabolites from oPLS-DA: Birth Length.

				Pearson’s Correlation	
	VIP score	Fold Change (Alc/Con)	q-Value	r-Value	*p*-Value
X-23639	2.968	1.38	0.1642	−0.49	0.0003
X-11795	2.756	2.48	0.0157	−0.47	0.0006
glycerophosphoinositol	2.505	1.06	0.6227	−0.36	0.0113
trigonelline (N′-methylnicotinate)	2.424	1.22	0.3079	−0.47	0.0006
1-palmitoleoyl-GPC (16:1)	2.418	1.36	0.2538	−0.38	0.0072
X-11880	2.389	0.58	0.0218	0.45	0.0010
1-oleoylglycerol (18:1)	2.377	1.14	0.5397	−0.37	0.0081
1-(1-enyl-palmitoyl)-2-arachidonoyl-GPC (P-16:0/20:4)	2.330	0.86	0.2669	0.43	0.0019
X-12117	2.313	0.92	0.7353	−0.31	0.0266
gamma-glutamylglutamate	2.303	1.30	0.2818	−0.28	0.0463
X−25855	2.287	1.13	0.5264	−0.29	0.0415
pregnanolone/allopregnanolone sulfate	2.279	1.03	0.9834	−0.31	0.0290
myristate (14:0)	2.275	0.97	0.9188	−0.32	0.0232
myo-inositol	2.275	1.05	0.8201	−0.33	0.0196
palmitoleate (16:1n7)	2.249	1.29	0.4536	−0.27	0.0581
glycerol 3-phosphate	2.235	1.13	0.6319	−0.38	0.0063
5alpha-pregnan-diol disulfate	2.205	1.02	0.7277	−0.33	0.0212
16-hydroxypalmitate	2.155	0.94	0.6319	−0.21	0.1450
2-linoleoylglycerol (18:2)	2.139	0.70	0.3825	−0.34	0.0144
3,5-dichloro-2,6-dihydroxybenzoic acid	2.137	0.94	0.9000	0.48	0.0004
3-hydroxymyristate	2.135	1.01	0.9188	−0.20	0.1640
pentose acid	2.104	1.21	0.7181	−0.32	0.0247
glutamate	2.089	1.03	0.7008	−0.33	0.0177
N-acetylputrescine	2.080	1.10	0.6438	−0.21	0.1410
ribitol	2.066	1.08	0.6654	−0.27	0.0596
X-21339	2.061	0.61	0.1244	0.36	0.0100
2-oleoylglycerol (18:1)	2.058	1.09	0.4511	−0.36	0.0104
1-(1-enyl-palmitoyl)-2-linoleoyl-GPC (P-16:0/18:2)	2.054	0.73	0.0264	0.38	0.0068
X-24951	2.041	0.61	0.0330	0.27	0.0565
X-11308	2.033	0.64	0.0578	0.31	0.0295

**Table 4:** Top 30 metabolites from oPLS-DA: Birth Weight.

				Pearson’s Correlation	
	VIP Score	Fold Change (Alc/Con)	q-Value	r-Value	*p*-Value
X-11795	2.859	2.48	0.0157	−0.53	0.0001
X-25855	2.744	1.13	0.5264	−0.44	0.0014
gamma-glutamylglutamate	2.724	1.30	0.2818	−0.39	0.0052
X-23639	2.606	1.38	0.1642	−0.40	0.0038
glycerol 3-phosphate	2.590	1.13	0.6319	−0.41	0.0032
1-oleoylglycerol (18:1)	2.563	1.14	0.5397	−0.44	0.0014
X-11880	2.550	0.58	0.0218	0.51	0.0001
X-11308	2.466	0.64	0.0578	0.48	0.0004
1-linoleoylglycerol (18:2)	2.406	0.69	0.3943	−0.41	0.0032
glycerophosphoinositol	2.394	1.06	0.6227	−0.39	0.0046
2-linoleoylglycerol (18:2)	2.368	0.70	0.3825	−0.42	0.0021
X-12117	2.355	0.92	0.7353	−0.34	0.0147
arabitol/xylitol	2.345	1.17	0.7973	−0.27	0.0555
glutamate	2.327	0.88	0.3825	−0.43	0.0019
5alpha-pregnan-diol disulfate	2.306	1.02	0.7277	−0.36	0.0104
1-palmitoleoyl-GPC (16:1)	2.261	1.36	0.2538	−0.33	0.0202
sphinganine-1-phosphate	2.254	1.21	0.9834	−0.34	0.0171
*N*-acetylputrescine	2.237	1.10	0.6438	−0.29	0.0421
glycine	2.221	1.04	0.6572	−0.29	0.0393
myo-inositol	2.218	1.05	0.8201	−0.33	0.0189
sphingosine-1-phosphate	2.204	1.08	0.8362	−0.26	0.0644
guanosine	2.191	1.07	0.8873	−0.22	0.1240
cotinine	2.179	2.80	0.0250	−0.50	0.0003
X-24951	2.168	0.61	0.0330	0.35	0.0125
*N*-1-methylinosine	2.163	1.02	0.9689	−0.30	0.0357
pregnanolone/allopregnanolone sulfate	2.161	1.03	0.9834	−0.28	0.0475
ribitol	2.159	1.08	0.6654	−0.27	0.0626
*N*-palmitoyltaurine	2.156	0.94	0.6572	−0.24	0.0946
1-arachidonylglycerol (20:4)	2.151	0.75	0.4413	−0.32	0.0214
X-11372	2.137	0.60	0.0218	0.40	0.0042

**Table 5:** Top 30 metabolites from oPLS-DA: Birth OFC.

				Pearson’s Correlation	
	VIP Score	Fold Change (Alc/Con)	q-Value	r-Value	*p*-Value
X-11795	2.898	2.48	0.0157	−0.47	0.0005
X-11880	2.647	0.58	0.0218	0.48	0.0004
X-11308	2.630	0.64	0.0578	0.51	0.0002
X-25855	2.569	1.13	0.5264	−0.47	0.0005
X-23639	2.560	1.38	0.1642	−0.37	0.0091
1-oleoylglycerol (18:1)	2.541	1.14	0.5397	−0.47	0.0006
1-palmitoleoyl-GPC (16:1)	2.472	1.36	0.2538	−0.41	0.0029
X-12117	2.431	0.92	0.7353	−0.49	0.0003
X-11372	2.382	0.60	0.0218	0.43	0.0017
X-24425	2.373	0.88	0.5130	0.55	0.0000
5alpha-pregnan-diol disulfate	2.308	1.02	0.7277	−0.38	0.0065
hydroxypalmitoyl sphingomyelin (d18:1/16:0(OH))	2.305	0.75	0.0218	0.35	0.0114
glycerol 3-phosphate	2.301	1.13	0.6319	−0.43	0.0018
gamma-glutamylglutamate	2.290	1.30	0.2818	−0.29	0.0415
N1-Methyl-2-pyridone-5-carboxamide	2.186	0.92	0.4536	0.35	0.0134
1-(1-enyl-palmitoyl)-2-arachidonoyl-GPC (P-16:0/20:4)	2.169	0.86	0.2669	0.38	0.0068
inosine	2.164	0.96	0.9621	−0.31	0.0305
2-linoleoylglycerol (18:2)	2.155	0.70	0.3825	−0.45	0.0011
arabitol/xylitol	2.148	1.17	0.7973	−0.28	0.0522
ribitol	2.140	1.08	0.6654	−0.35	0.0130
1-linoleoylglycerol (18:2)	2.134	0.69	0.3943	−0.42	0.0023
X-11381	2.132	0.86	0.6531	0.48	0.0004
cotinine	2.125	2.80	0.0250	−0.38	0.0071
4-hydroxychlorothalonil	2.101	0.60	0.0840	0.48	0.0005
1-stearoyl-2-docosahexaenoyl-GPC (18:0/22:6)	2.101	0.91	0.7008	0.42	0.0021
guanosine	2.087	1.07	0.8873	−0.24	0.0916
glycine	2.081	1.04	0.6572	−0.36	0.0102
sphingomyelin (d18:2/18:1)	2.064	0.63	0.0494	0.39	0.0056
sphingomyelin (d18:2/23:1)	2.053	0.71	0.0264	0.42	0.0022
3,5-dichloro-2,6-dihydroxybenzoic acid	2.053	0.94	0.9000	0.43	0.0018

## Data Availability

Not applicable.

## Supplementary Materials

The following are available online at: https://www.mdpi.com/article/10.3390/nu14245367/s1, Figure S1: Principal Component Analysis (PCA) of 782 Metabolites by Infant Sex; Figure S2: Principal Component Analysis (PCA) Biplot; Figure S3: Principal Component Analysis (PCA) Loading Plot; Figure S4: Principal Component Analysis (PCA) Scree Plot; Figure S5: oPLS-DA Plots for (a) infant birth length, (b) infant birth weight, (c) infant head circumference (OFC); Table S1: Top 30 Metabolites Loading on PC1; Table S2: Top 30 Metabolites Loading on PC2.

## Abbreviations

Drinks per drinking day = DDD; false discovery rate = FDR; fetal alcohol spectrum disorders = FASD; occipitofrontal circumference = OFC; partial least square discriminant analysis = PLD-DA; PAE = prenatal alcohol exposure; principal component analysis = PCA; tricarboxylic acid = TCA; variable importance projection = VIP.
