# Supplementary material for: Untargeted Metabolome Analysis of Alcohol-Exposed Pregnancies Reveals Metabolite Differences That Are Associated with Infant Birth Outcomes
Source: Nutrients. 2022 Dec 17;14(24):5367. doi: 10.3390/nu14245367 (PMC9786146; doi:10.3390/nu14245367)
Supplement: Supplementary file 1 [file nutrients-14-05367-s001.zip › nutrients-2052899-supplementary.pdf]

Untargeted Metabolome Analysis of Alcohol-Exposed Pregnancies Reveals Metabolite Differences that Are Associated with Infant Birth Outcomes

Supplemental Material

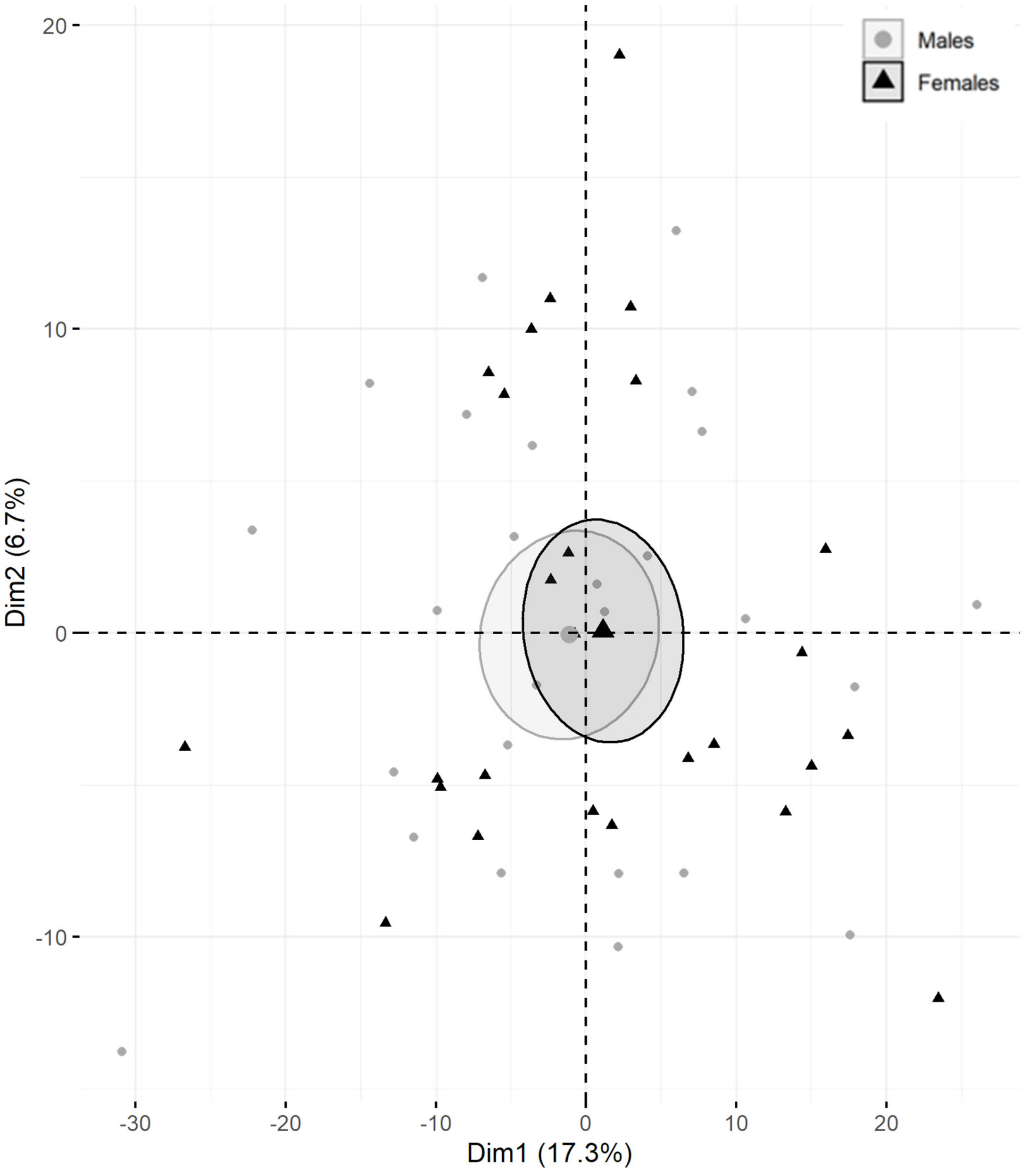

Figure S1. Principal Component Analysis (PCA) of 782 Metabolites by Infant Sex (*n*=52). Gray circles denote male offspring. Black triangles denote female offspring.

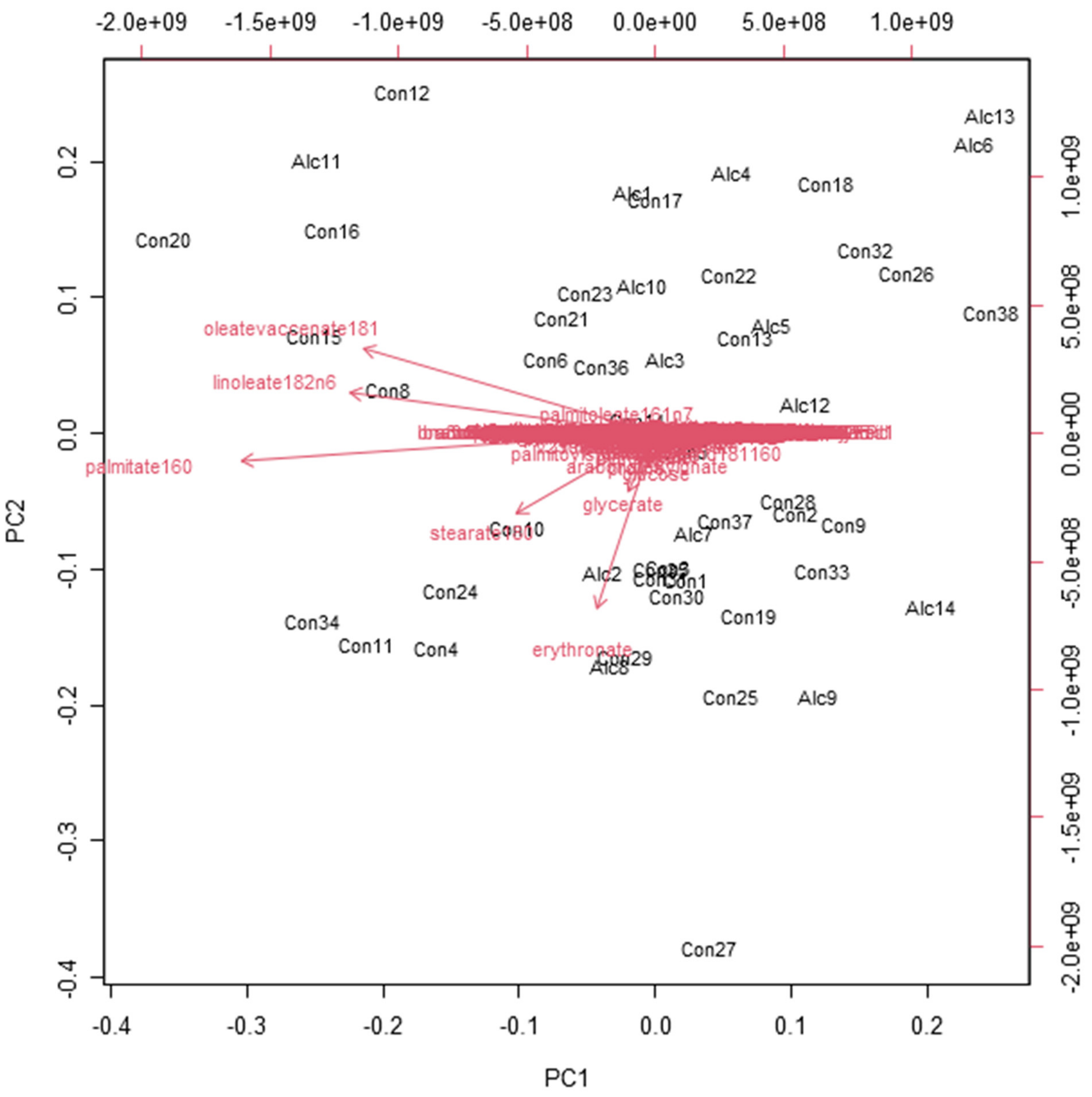

Figure S2. Principal Component Analysis (PCA) Biplot

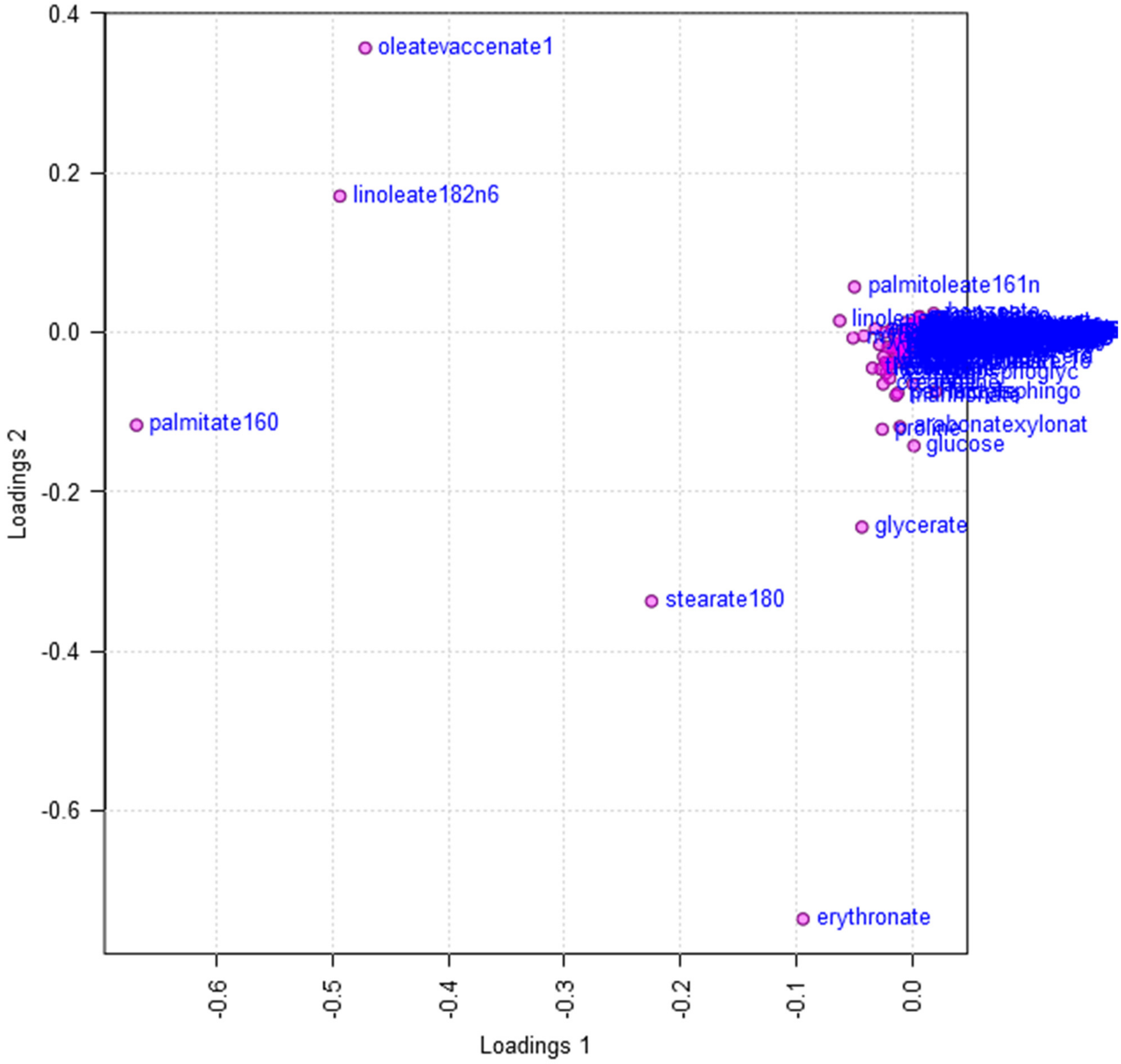

Figure S3. Principal Component Analysis (PCA) Loading Plot

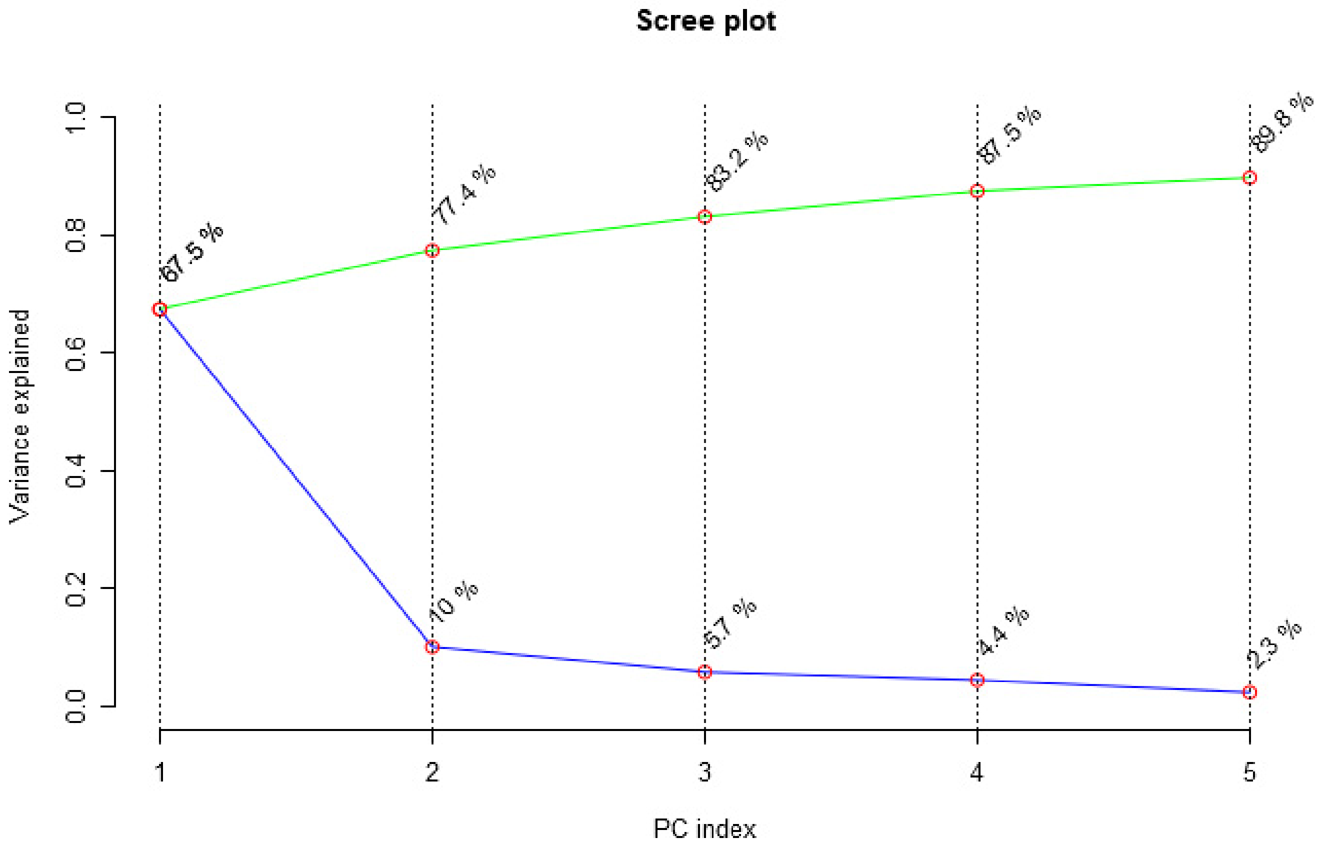

Figure S4. Principal Component Analysis (PCA) Scree Plot

a) Infant Birth Length

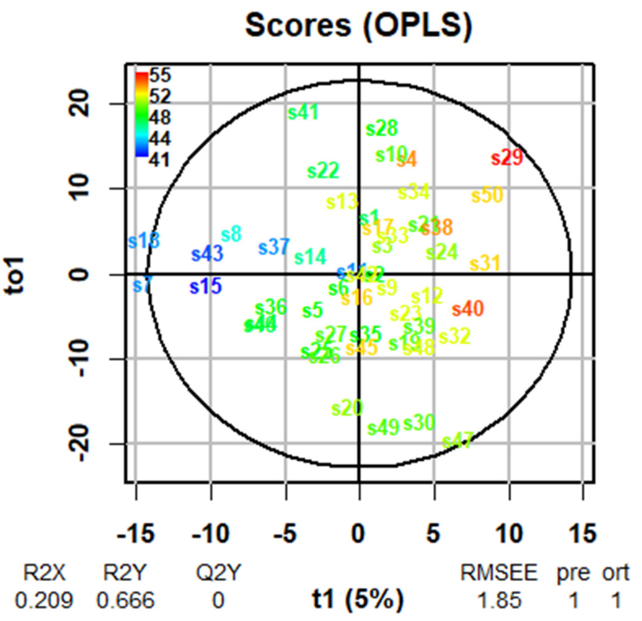

b) Infant Birth Weight

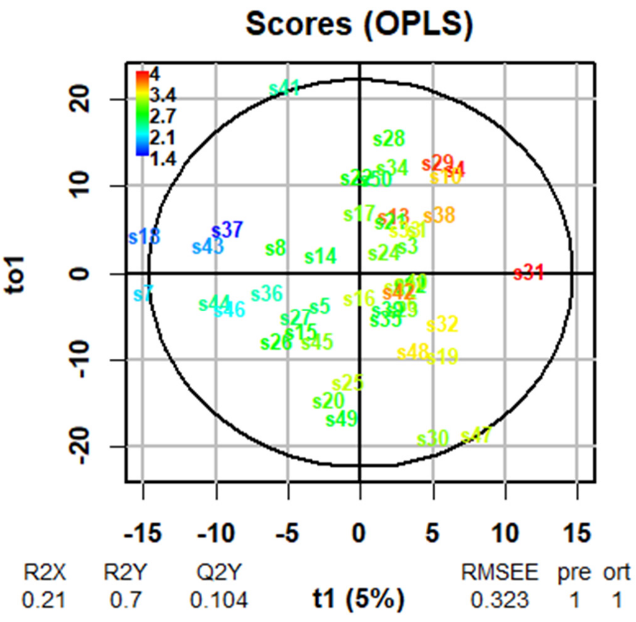

c) Infant Birth Head Circumference

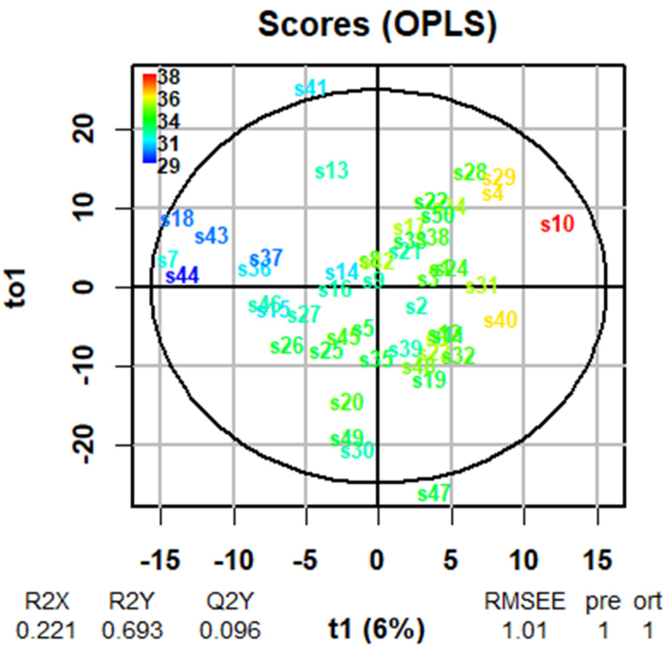

Figure S5. oPLS-DA Plots: a) infant birth length; b) infant birth weight; c) infant birth head circumference (OFC)

Table S1. Top 30 Metabolites Loading on PC1

Metabolite	PC1 Loading
palmitate (16:0)	-0.66919
linoleate (18:2n6)	-0.49372
oleate/vaccenate (18:1)	-0.47183
stearate (18:0)	-0.22477
erythronate	-0.094114
linolenate [alpha or gamma; (18:3n3 or 6)]	-0.062449
myristate (14:0)	-0.05094
palmitoleate (16:1n7)	-0.049739
glycerate	-0.043289
13-HODE + 9-HODE	-0.041659
threonate	-0.034225
arachidonate (20:4n6)	-0.031866
1-palmitoyl-2-oleoyl-GPC (16:0/18:1)	-0.028133
isoleucine	-0.027176
proline	-0.025723
creatine	-0.024977
leucine	-0.02468
5-oxoproline	-0.022927
laurate (12:0)	-0.022821
2,3-diphosphoglycerate	-0.022443
1-palmitoyl-2-linoleoyl-GPC (16:0/18:2)	-0.020905
valine	-0.020764
lactate	0.020177
X-21796	-0.019337
creatinine	-0.019283
benzoate	0.018692
1-palmitoyl-GPC (16:0)	-0.017483
choline	-0.017385
margarate (17:0)	-0.016828
phosphate	-0.015233

Table S2. Top 30 Metabolites Loading on PC2

Metabolite	PC2 Loading
erythronate	-0.73632
oleate/vaccenate (18:1)	0.3564
stearate (18:0)	-0.33767
glycerate	-0.24458
glucose	-0.14277
proline	-0.12188
arabonate/xylonate	-0.11883
palmitate (16:0)	-0.11669
mannonate	-0.079249
palmitoyl sphingomyelin (d18:1/16:0)	-0.076379
lactate	-0.074887
betaine	-0.0654
creatine	-0.065166
X-21796	-0.057841
2,3-diphosphoglycerate	-0.051236
isoleucine	-0.046392
creatinine	-0.046322
threonate	-0.045161
valine	-0.039162
1-palmitoyl-GPC (16:0)	-0.039058
p-cresol sulfate	-0.038733
adenosine 5'-monophosphate (AMP)	-0.038565
5-oxoproline	-0.037776
caproate (6:0)	-0.033666
3-phosphoglycerate	-0.032251
leucine	-0.0309
carnitine	-0.026464
phosphate	-0.023961
arachidate (20:0)	-0.023829
alanine	-0.022703
